# Dual Fluorescence in Glutathione-Derived Carbon Dots Revisited

**DOI:** 10.1021/acs.jpcc.1c10478

**Published:** 2022-01-26

**Authors:** Yadolah Ganjkhanlou, J.J. Erik Maris, Joris Koek, Romy Riemersma, Bert M. Weckhuysen, Florian Meirer

**Affiliations:** Inorganic Chemistry and Catalysis, Debye Institute for Nanomaterials Science, Utrecht University, 3584 CG Utrecht, The Netherlands

## Abstract

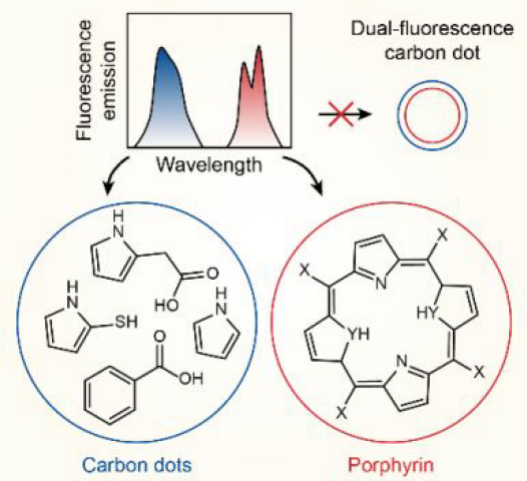

Dual-fluorescence carbon dots have great potential as nanosensors in life and materials sciences. Such carbon dots can be obtained via a solvothermal synthesis route with glutathione and formamide. In this work, we show that the dual-fluorescence emission of the synthesis products does not originate from a single carbon dot emitter, but rather from a mixture of physically separate compounds. We characterized the synthesis products with UV–vis, Raman, infrared, and fluorescence spectroscopy, and identified blue-emissive carbon dots and red-emissive porphyrin. We demonstrate an easy way to separate the two compounds without the need for time-consuming dialysis. Understanding the nature of the system, we can now steer the synthesis toward the desired product, which paves the way for a cheap and environmentally friendly synthesis route toward carbon dots, water-soluble porphyrin, and mixed systems.

## Introduction

Carbon dots (CDs) are fluorescent carbon-based nanoparticles with tunable and functional photoluminescence.^1,2^ Since their serendipitous discovery during carbon nanotube purification in 2004,^3^ they have gained significant interest for application as a new generation light source,^4−6^ solution-based chemo-sensors for detecting chemicals and metal cations,^2,7−11^ and nanoprobes for mapping biological systems.^2,7−11^ Moreover, they have shown great potential to be applied directly or in hybrid materials for photocatalytic and electrocatalytic applications.^12−16^ CDs are generally regarded as environmentally friendly, nontoxic material because the synthesis precursors are relatively abundant, harmless, and environmentally friendly and the synthesis does not involve extreme reaction conditions nor produces harmful waste.^17−21^ This motivates their use outside the academic research lab in industrial and consumer applications. An example being their use as sensors for the detection of dangerous chemicals, for the detection of reaction products in high-throughput catalyst testing, or even to map the heterogeneity in the chemical composition of porous materials. All these applications revolve around the control over their tunable^22^ and functional photoluminescence (PL) properties.

A thorough understanding of CD synthesis and its effect on the PL properties are essential to obtain the desired sensor behavior. Briefly, CDs have been synthesized via both top-down and bottom-up approaches. In the top-down synthesis approach, larger carbon structures, such as graphite, are broken down into smaller CDs, while in the bottom-up synthesis approach, small carbonaceous precursors are heated to obtain CDs through carbonization and graphitization.^23^ CDs are on the order of ten nanometers in size and consist of both amorphous and crystalline carbon structures with sp^2^–sp^3^ hybridization. The carbon structures contain primarily oxygen and nitrogen heteroatoms and the exact composition is heavily dependent on the synthesis precursor and synthesis conditions.^23^

The PL properties of CDs are of paramount importance for their application. Four origins of PL emission in CDs have been identified: the carbon core state luminescence from a conjugated π-system with quantum confinement, the surface state luminescence from interaction between the core state and surface groups, embedded fluorophores in the carbon matrix, and the cross-link enhanced emission effect.^24−26^ It has traditionally been reported that the core and surface states are the origin of PL emission in CDs. However, recent studies have demonstrated that embedded fluorophores are responsible for PL emission with high quantum efficiency. These embedded fluorophores are commonly found in CDs prepared by bottom-up approaches.^27^ In some systems, the interaction of surface states with the CD’s local environment results in a measurable response in the fluorescence intensity and/or wavelength. Such sensing capacities are especially powerful when present for one of the two bands of dual-fluorescence CDs. These nanoparticles have two distinct PL emission bands that both originate from the same CD, which allows the extraction of sensory information from one band as well as a local CD concentration from the other. Such a ratiometric approach is advantageous, because the acquired sensory data is independent of the CD concentration. Macairan et al. and Wang et al. recently reported a procedure to synthesize dual-fluorescence CDs based on glutathione (GSH) solved in formamide (FA).^7,28,29^ When excited at 405 nm, they emit a broad blue PL emission band with a maximum emission around 460–490 nm and a narrow red emission band at 650–680 nm. The system was reported to be suitable for mapping cells and intracellular parameters of the environment, such as the local pH, and consequently, it may be suitable for mapping of heterogeneous catalysts and other functional porous materials.

To correctly use the GSH/FA CD system as a nanoprobe, we need to know the mechanism behind the blue and red PL emission to understand their response to the environment. In this work, we show that the blue- and red-emitting species are two physically separable compounds, and we reveal their chemical identity. Moreover, we demonstrate an easy and environmentally friendly separation method to purify the obtained synthesis products. After the components of the GSH/FA CD system have been identified, we demonstrate control over the synthesis via acid and base catalysis, which we employ to obtain the desired products in increased yield. Altogether, this paves the way for a successful use of this CD system as a nanosensor in porous solids and other sensing applications.

## Methods

### Synthesis and Dialysis

The carbon dots (CDs) were synthesized following an adaptation from ref (28). Reduced L- glutathione (GSH) was dissolved in a formamide (FA) at room temperature. The mixture was poured in a Teflon-lined autoclave (Parr 4749) and was heated in solvothermal conditions to a temperature of 180 °C for 3 or 18 h. The obtained solution was filtered through a 0.22 μm filter and centrifuged at 5000 rpm for 5 min to remove large aggregates. The supernatant solution was dialyzed using a 3.5 kDa dialyzing membrane (D-Tube Dialyzer Maxi, MWCO 3.5 kDa, Merck) for 1 week, while the water was replaced daily. The progress of the separation was followed via the luminescence of the dialysate excited with a 405 nm laser pointer (5 mW). Approximately 7 days of dialysis turned out to be sufficient to reduce the concentration of luminescent species in the dialysate below a level observable by the eye. Additional samples with other solvents (H_2_O and *N*,*N*-dimethylformamide) or in acidic and basic environments have been prepared (Table S1).

### Purification by Kaolinite

Red-emissive products were separated from blue-emissive ones via purification with kaolinite. Ten milliliters synthesis product solution (before dialysis) was mixed with 1 g kaolinite and 0.1 mL HCl (37%). The mixture was left for 10 min, and the kaolinite particles were sedimented by centrifugation at 5000 rpm for 5 min. The supernatant containing the blue-emissive product was taken. To remove blue-emissive product impurities from the sediment, it was washed with 20 mL methanol and separated from kaolinite by centrifugation (5000 rpm, 5 min). The methanol washing step was repeated once more. To release the red-emissive products from the sediment, it was dispersed in a solution of 10 mL of 7 M ammonia in methanol for 10 min before the kaolinite particles were centrifuged down (5000 rpm, 5 min) and the supernatant containing the red-emissive product was taken. Finally, this solution was dried at room temperature under N_2_ flow (20 mL/min), and a dark green powder was obtained and stored in the freezer. The obtained powder can be easily solved in water for further use.

### Characterization

**Figure 1:**
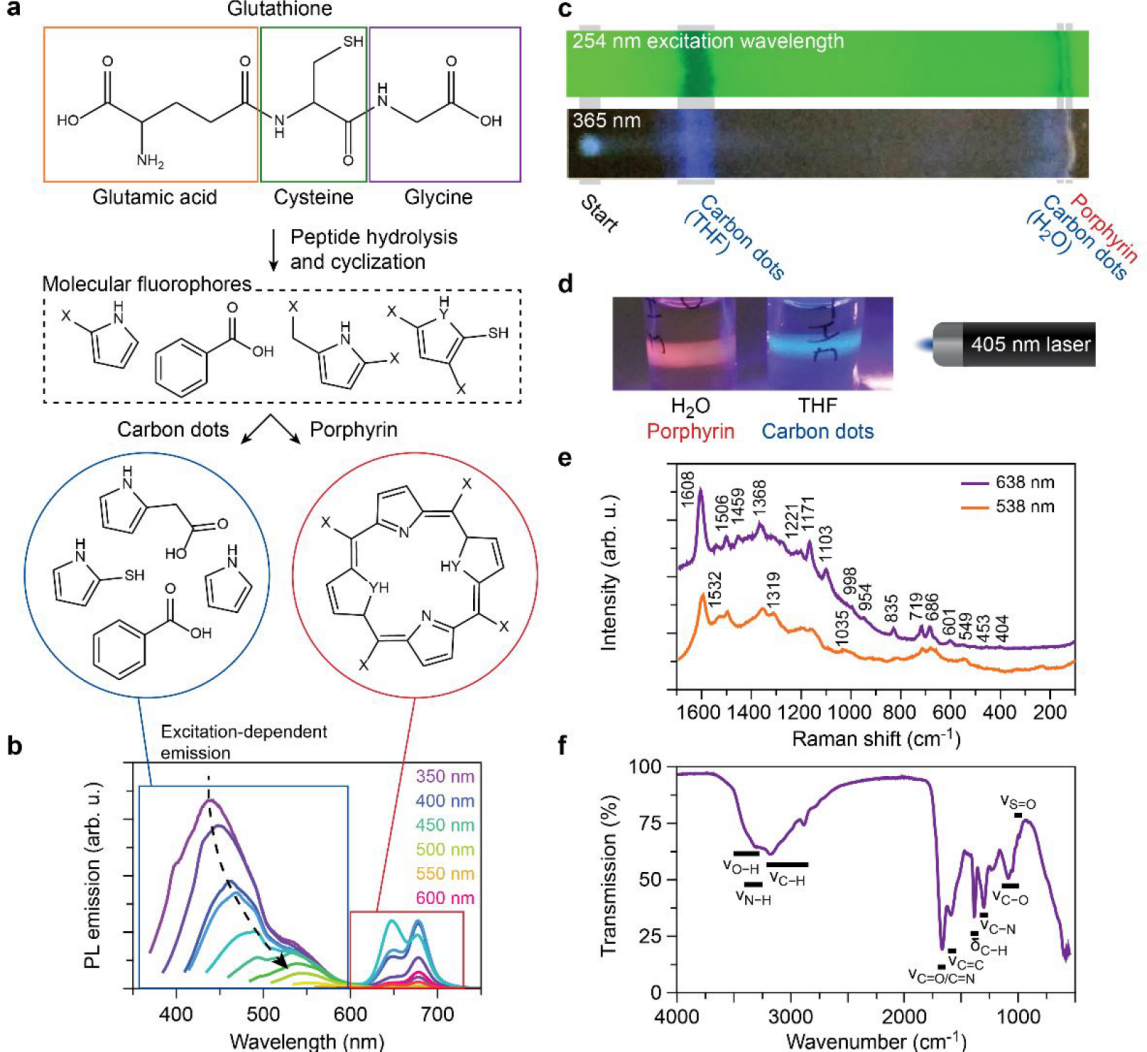
(a) Schematic of the solvothermal synthesis of carbon dots and porphyrin from glutathione in formamide. After peptide hydrolysis and cyclization, small molecular fluorophores are formed in solution. These fluorophores react further to carbon dots and porphyrin. X represents polar functional groups such as COOH, OH, SH, phenol, and glutamate and YH is S or NH. Please note that the structures of the fluorophores are examples, and many more variations are possible. (b) Photoluminescence (PL) emission spectra as a function of the excitation wavelength after 3 h of solvothermal synthesis and dialysis of the product with a 3.5 kDa membrane (GSH-FA-3h). The carbon dots have an excitation-dependent emission (400–600 nm) and the porphyrin has an excitation-independent emission in the red (600–750 nm). (c) Thin-layer chromatography of the GSH-FA-3h sample using 50/50 (v/v) water/THF mixture as the mobile phase. Three bands with separable compounds are observed (top), which were identified as carbon dots with blue emission and porphyrin with red emission. (d) PL emission of GSH-FA-3h dissolved dried powder in different solvents excited with a 405 nm laser pointer. In water, the emission is red, indicating that the porphyrin is water-soluble and hydrophilic, while the emission is blue in THF, demonstrating amphiphilic nature of the carbon dots. (e) Raman spectrum of the GSH-FA-3h sample recorded with 538 and 638 nm excitation wavelength is similar to that of hematoporphyrin. (f) Infrared transmission spectrum of the GSH-FA-3h sample indicates the formation of conjugated carbon structures with oxygen and nitrogen functional groups.

UV–vis spectra were recorded using a UV-Cary 200 spectrophotometer. Photoluminescence emission spectra in Figure 1b were recorded with a Jasco spectrofluorometer (FP 8300) at 200 nm/min. The photoluminescence spectra in all other figures were recorded using AvaSpec-ULS2048CL-EVO as a spectrophotometer and AvaLight-HPLED-405 as a light source with a wavelength of 405 nm. All emission spectra were recorded at 405 nm excitation wavelength unless otherwise noted. Fourier-transform infrared (IR) spectra have been measured in attenuated total reflectance mode by a PerkinElmer 2000 instrument on a droplet of CDs with a concentration of 1 g/L. X-ray diffractograms (XRD) have been recorded by a D2 Bruker diffractometer in the 2θ range of 10–40° using a Co Kα source and slit size of 1 mm. For XRD measurements, 1 mL aqueous solution of CDs with a concentration of 1 g/L was dried on a glass substrate, which was repeated until a layer had formed. The same dried samples were used for Raman spectroscopy measurements. Raman spectra were acquired using a Horiba XPlora microscope equipped with 638 and 532 nm lasers. The power at the sample was 0.22 mW for the 638 nm laser and 0.53 mW for the 532 nm laser. All measurements were done with a 1200 l/mm grating and an exposure time of 100 s. Because porphyrin absorbs light at 638 nm, the signal from the porphyrin is enhanced as a result of resonance Raman scattering. For atomic force microscopy (AFM) measurements, a droplet of diluted solution (C ≈ 10 mg/L) of CDs was dried on a circular glass coverslip and the measurements were performed on a Bruker Multimode 8 in peakforce/Scanasyst mode with ScanAsyst-air probes. The scanned area was about 2 × 2 μm for the recorded AFM images. The NanoScope Analysis V1.80 software was used to construct a topography map from the AFM images. Transmission Electron Microscopy (TEM) images were acquired on a FEI Talos L120C electron microscope. Five microliters of as-prepared sample solution drop-casted on a copper Formvar grid and were left to dry in the air. The optical images of dried sample droplet on glass coverslip were measured using the ZEISS AXIO Zoom.V16 microscope using 2.3× objective.

## Results and Discussion

Solvothermal heating of GSH in FA results in the generation of fluorescent synthesis products. GSH is a peptide comprised of three amino acids that hydrolyses and cyclizes at elevated temperatures forming fluorescent reaction intermediates (Figure 1a). These intermediates were observed in the dialysate through a 3.5 kDa membrane, confirming their size in the range of small molecules (Figure S1a). These molecular fluorophores can be detected during the initial steps of heating (*T* = 160 °C, Figure S1b). Over time, these species react further to the CD products. We have observed that the synthesis products after dialysis have two distinct emission bands, which were ascribed by Macairan et al. to dual emissive CDs (Figure 1b).^28^ These authors have attributed the 400–600 nm PL emission to a core state and the 600–750 nm emission to a molecular surface state but did not find evidence for energy transfer between the states. Our findings show that these emissive states originate from a blue-emitting CD and a red-emitting molecular fluorophore called porphyrin (400 nm excitation, Figure 1a–b), revealing that the two states are not just physically distinct but rather physically separated.

We have observed that the blue PL emission is excitation-dependent, while the red emission is excitation-independent (Figure 1b). Excitation-dependent emission is typical for CDs, which shows that the blue-emitting compounds are CDs. The origin of excitation-dependent emission is a matter of conflict in literature, but it is generally accepted that the behavior can be explained by a heterogeneous composition of fluorescent centers or complex energy levels.^25,30−32^ Here, only the subpopulation of excited states that are efficiently populated at the excitation wavelength contribute to the PL emission, which results in a shift of the emission with excitation wavelength. This mechanism has been attributed to embedded molecular fluorophores in the CDs, aggregation of CDs, and the presence of different types of CDs.^25,30−32^ It was found that the red emission is excitation independent, which indicates that it is related to a specific molecular fluorophore. This fluorophore is not removed during dialysis with a 3.5 kDa membrane (Figure S1a). Therefore, it can be concluded that the red-emissive molecule either is embedded in the CDs or is a macromolecule. To address this question, we performed thin-layer chromatography (TLC) using a 50/50 (v/v) water/THF mixture as a mobile phase. The separated analytes are visible as a dark band upon excitation of the TLC plate with 254 nm ultraviolet (UV) light (Figure 1c). We used 365 nm excitation to record the PL color and identify the separated species. We find three bands: the first broad band is from analytes running with the THF mobile phase and contains mainly blue-emitting CDs; the other two narrow bands originate from the water mobile phase and contain blue-emitting CDs and red-emitting macromolecules. TLC shows that red emissive products are not embedded in the CDs as they can be separated from the blue-emitting species and must be a macromolecule. The CDs are amphiphilic and dissolve in both water and THF, with a larger affinity for the THF phase, while the macromolecule is hydrophilic and only dissolves well in the water mobile phase. Indeed, solvent extraction with water and THF results in the expected separation (Figure 1d). Red emission was observed in water, while blue emission was observed in THF when excited with 405 nm laser light. The presence of sediments confirmed the poor solubility of the red-emitting macromolecule in THF.

We identified the macromolecule as a water-soluble porphyrin using UV–vis, Raman, and infrared (IR) spectroscopy. These synthesis products cannot be identified from the X-ray diffraction pattern since they tend to form an amorphous layer upon drying (Figure S2). However, the product obtained has clear Soret and Q bands in the UV–vis absorption spectrum, which are characteristic for porphyrin molecules. Moreover, the recorded Raman spectra (Figure 1e) are to a large extent similar to the spectra of hematoporphyrin complexes, and their band assignment can be found in Berjot et al.^33^ We found many narrow bands, which indicate the presence of a molecular species. We did not observe the D and G bands in the Raman spectrum, which are related to the degree of graphitization in CDs, and the features of porphyrin dominated.^28,34^ The narrow band at 1600 cm^–1^ could be falsely assigned to the carbon G band but is actually associated with the intensity of the bands around 600–800 cm^–1^, which are due to ring deformations of the porphyrin. Finally, IR spectroscopy confirmed the formation of conjugated carbon structures with oxygen and nitrogen functional groups.^35−37^ The assigned functional groups are listed in Figure 1f and Table S2. The most characteristic functional groups are the N–H at 3320 cm^–1^, C=N or C=O in amide around 1673 cm^–1^, and C=C in the aromatic ring at 1591 cm^–1^.^35−37^ On top of that, the IR spectra have high similarity to water-soluble porphyrin molecules and building blocks (e.g., proline).^38^ Hence, all these spectroscopic features are very similar to CDs synthesized from natural carbon sources rich in chlorophyll-based porphyrin, which further supports our assignment.^39,40^

**Figure 2:**
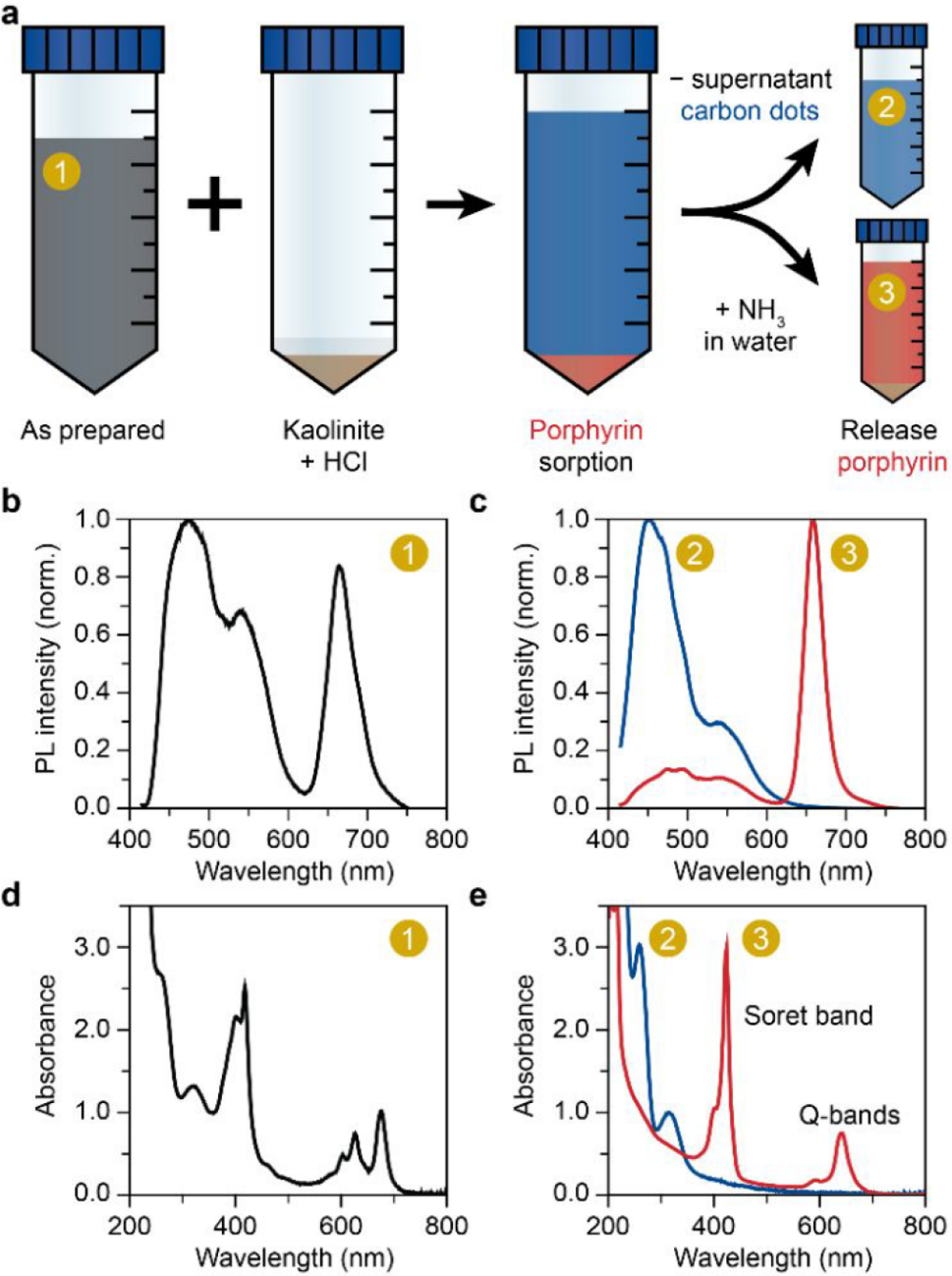
(a) Schematic representation of the kaolinite purification method. The synthesis reaction mixture containing carbon dots, porphyrin, and residual molecular fluorophores (1) were separated by adsorption of the porphyrin and residual fluorophores on the kaolinite (GSH-FA-3h). The supernatant containing the carbon dots (2) was taken and the porphyrin and residual fluorophores were released in basic ammonia solution (3). (b–c) The normalized photoluminescence (PL) emission spectra prior to (b) and after (c) purification. The numerals (1–3) indicate the respective solution as indicated in (a). (d–e) UV–vis absorption spectra prior to (b) and after (c) purification. The numerals (1–3) indicate the respective solution as indicated in (a). The Soret and Q-band(s) of the porphyrin are indicated after purification.

Next, we have developed an alternative purification method to dialysis by using kaolinite as a sorbent, which is a faster and more efficient way to separate porphyrin from CDs and most of the small molecules. Separation facilitates detailed analysis of the individual components as well as the use in applications requiring either CDs or porphyrin. Porphyrin molecules, especially the water-soluble derivatives, have a high affinity to be adsorbed in the layered structure of kaolinite.^41^ For separation, we stirred the synthesis product mixture with kaolinite in acidic conditions, which promotes the sorption of porphyrin (Figure 2a). Then, we sedimented the kaolinite particles by centrifugation: the porphyrin partitioned in the kaolinite sediment, while the CDs remained in solution. This allowed us to harvest the CDs from the supernatant solution. We then removed the remaining blue-emitting impurities from the kaolinite by washing it two times with methanol. Finally, we released the porphyrin from the sediment by addition of a basic methanol solution. A dark green powder was obtained after drying under nitrogen flow. The last step can also be performed with an aqueous solution; however, we used methanol as the main solvent, since it is easier to dry the obtained solution.

We could demonstrate the quality of separation of the kaolinite purification method via fluorescence and UV–vis spectroscopy (Figure 2b–e). The PL spectral features we ascribed to CDs and porphyrin were well separated after kaolinite purification (Figure 2c). However, we find low intensity PL around 400–600 nm in the porphyrin fraction, which disappears after further purification with THF, indicating that this signal stems from residual molecular fluorophores. In the UV–vis spectrum of the porphyrin fraction, absorption bands at 400 nm and a series of absorption bands around 620 nm are present (Figure 2e). The band at 400 nm is the well-known Soret band, which is insensitive to the functional groups of the porphyrin and is absent only when porphyrin macrocyclic conjugation is disrupted.^42^ The series of bands around 620 nm are the Q-band absorptions. They are often observed between 500–650 nm but usually below 600 nm. A change in the functional groups of porphyrin, e.g., due to a change in pH, can cause shifts in the Q-bands, while the Soret band remains unaffected.^42^ This is illustrated by the change in the Q-bands prior to (Figure 2c) and after purification (Figure 2e). Altogether, we can conclude that the observed red-emissive macromolecule is indeed porphyrin.

**Figure 3:**
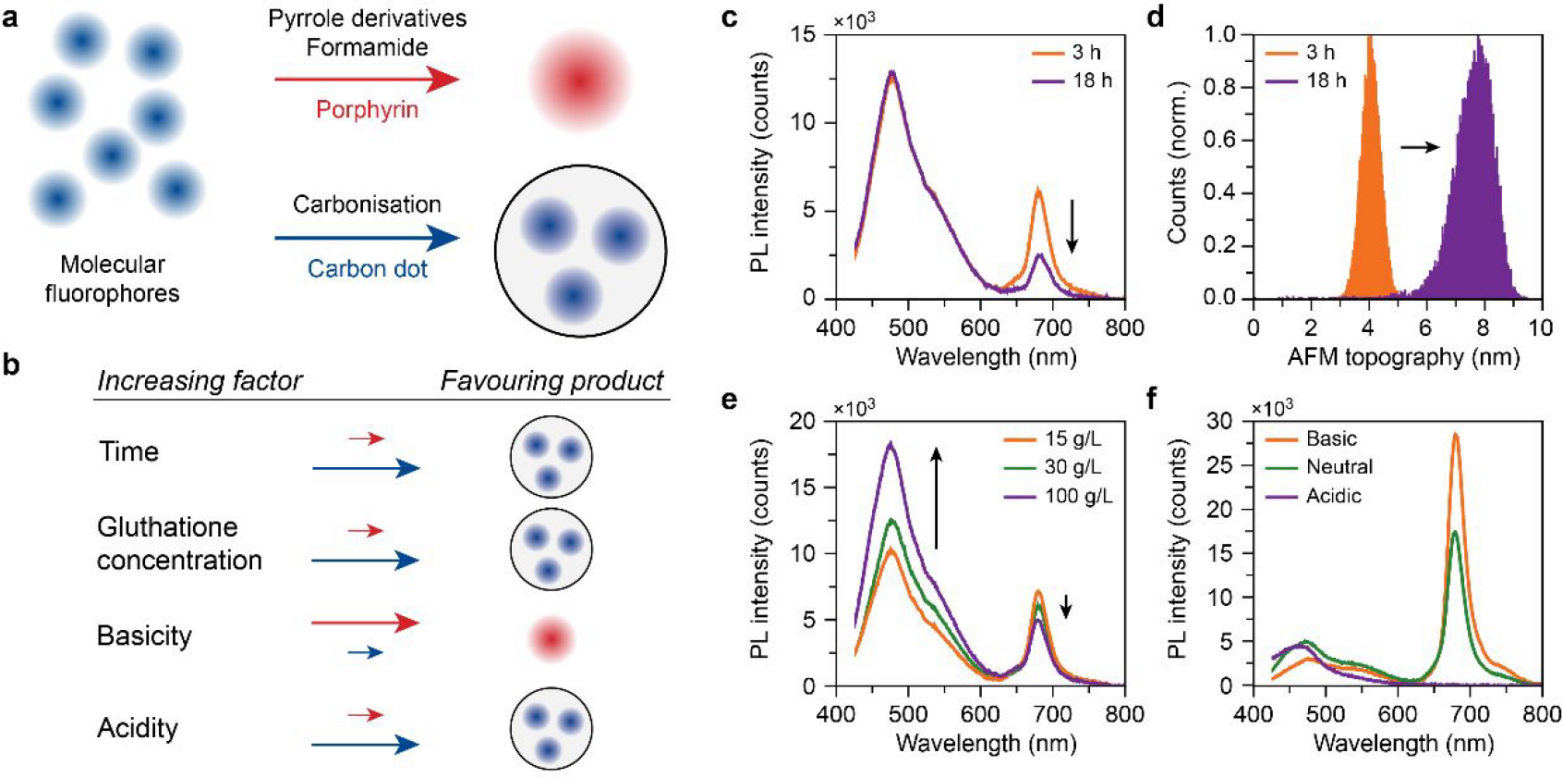
(a) Simplified scheme of the two competing reactions during the solvothermal synthesis of porphyrin and carbon dots from glutathione-condensation and -cyclization products. (b) Table of the favored product (right) as a function of increased factor during synthesis (left) following the reaction scheme in (a). Other factors were held constant with respect to the reported synthesis conditions in the methods. The presence of the favored product was derived from the photoluminescence (PL) emission spectra, where emission in the 400–600 nm range indicates the presence of carbon dots and unreacted molecular fluorophores, while emission in the 600–750 nm range marks the presence of porphyrin. (c–d) Normalized PL emission spectra (c) and atomic force microscopy (AFM) topography histogram (d) of the nonpurified synthesis products as a function of increasing reaction time (200× diluted in pH = 7.3 phosphate buffer; GSH-FA-3h and GSH-FA-18h). (e) Normalized PL emission spectra of the nonpurified synthesis products as a function of increasing glutathione concentration during synthesis (200× diluted in pH = 7.3 phosphate buffer; GSH-l-FA-3h, GSH-FA-3h, and GSH-FA-3h). (f) Normalized PL emission spectra of the nonpurified synthesis products as a function of synthesis pH (200× diluted in pH = 7.3 phosphate buffer; GSH-FA-5 min-basic, GSH-FA-5 min, and GSH-FA-5 min-acidic).

During the GSH/FA solvothermal synthesis, two main synthesis pathways are in competition (Figure 3a). The molecular fluorophores formed from GSH can either react with formamide to form porphyrin or carbonize to CDs. During the carbonization, the molecular fluorophores are embedded into amorphous carbon, giving the CDs their fluorescent properties. Indeed, the PL emission of the molecular fluorophores is almost identical to that of the CDs (Figure 2c). Moreover, the UV–vis absorption spectrum of the CD fraction solely comprises absorption in the UV range as would be expected for small molecular fluorophores (Figure 2e). These molecular fluorophores are formed in an early stage of the reaction (Figure S3).^24^ We believe that a major part of these species are pyrrole derivatives, which are known to form by heating some of the amino acids^43,44^ and have a PL emission spectrum similar to pyrrole. It is these pyrrole derivatives together with aldehydes that can condense into porphyrin molecules in the Rothemund condensation.^45^ Formamide plays a crucial role in the synthesis, because we did not obtain porphyrin in the GSH solvothermal synthesis in water and DMF (Figure S4). These results suggest that formamide can take up the role of an aldehyde due to its similar chemical structure and condense with pyrrole derivatives to porphyrin. Remarkably, we found that the individual amino acids in GSH do not form porphyrin (Figure S5). Moreover, a mixture of the amino acids including glycine, cysteine, and glutamic acid do not yield porphyrin either. This suggests that the full GSH peptide is required to form the pyrrole porphyrin precursors, and we speculate that cyclization occurs in the center of the GSH leading to pyrrole derivatives with two functional groups. This process appears to be universal as we have found a report of CDs synthesized from natural pulp-free lemon juice containing GSH, which have porphyrin spectroscopic signatures in both absorbance and PL emission.^46^

We investigated the effect of various synthesis conditions, as summarized in Figure 3b. A longer reaction time favored carbonization, which was marked by the relative decrease in porphyrin PL emission in the 600–750 nm range (Figure 3c). Macairan et al. concluded that a longer reaction time in the GSH/FA CD synthesis resulted in more C–N and C=N bonds measured with X-ray photoelectron spectroscopy (XPS), which indicate increased carbonization of the CDs.^28^ Also for other synthesis methods, a longer reaction time has been linked to a higher degree of carbonization.^25^ We observed with atomic force microscopy (AFM) that the size of the CDs increases from roughly 4 to 8 nm with reaction times from 3 to 18 h (Figure 3d). The size distribution of the CDs is narrow and well-defined, which is in line with a nucleation and growth mechanism reported previously.^25^ Porphyrin is below the detection limit of AFM as the planar molecule is known to arrange flat on the substrate surface. Transmission electron microscopy confirms the CD size and, in combination with optical microscopy, shows that the porphyrin self-assembles into dendritic structures at high concentration (Figure S6). The formation of porphyrin occurs mostly in the first 10 min of the reaction, monitored via the PL emission at various synthesis times (Figure S3a–b). As the reaction progresses, the relative abundance of porphyrin decreases, and the carbonization pathway dominates. A higher GSH concentration during the solvothermal synthesis also favors CD formation, again marked by the decrease of the porphyrin PL emission in the 600–750 nm domain (Figure 3e). Altogether, this results in a higher probability for nucleation, which leads to a higher CD concentration.

To further investigate the mechanism of porphyrin and CD formation, the synthesis was performed in acidic and basic conditions. Carbonization occurs rapidly in the acidic environment and no porphyrin PL emission was observed in the reaction mixture (Figure 3f). Conversely, basic reaction conditions favor porphyrin as main synthesis products because of the low carbonization rate. As discussed before, we believe that pyrrole derivatives are the precursor for porphyrin in this reaction. In accordance with the literature, the aromatization,^47^ condensation,^48^ and pyrrole formation^49^ are all favored in alkaline environments, while in acidic condition the dehydration and degradation of organic compounds are favored.^47,50^ In particular, chemical dehydration carbonizes the products and decreases the H/C and O/C ratio.^47,50^ We conclude that in an acidic environment side reactions are dominant, such as dehydration, which inhibits small molecule condensation and porphyrin formation.

In addition to blue-emitting fluorophores, which have a maximum PL emission around 460 nm, an additional emission band around 550 nm is intensified in a neutral and basic environment (Figure 3f). In accordance with literature, fluorophores with single aromatic rings (e.g., citrazinic acid) usually have blue emission, while those with two or more rings have green emission (e.g., hydroxy-1*H*-pyrrolo[3,4-*c*]pyridine-1,3,6(2*H*,5*H*)-trione (HPPT)).^27,51−53^ Therefore, we conclude that in neutral and basic reaction conditions, in addition to condensation reaction forming porphyrin molecules, the formation of polycyclic aromatic fluorophores is also possible. The high abundance of pyrrole species in basic synthesis conditions increases the probability for further reaction to polycyclic aromatic dyes, marked by the 550 nm PL emission (Figure 3f). Both the lower carbonization rate as well as the higher formation rate of pyrrole derivatives result in higher selectivity toward porphyrin. Thus, by steering the competition between carbonization and porphyrin formation, the desired product(s) can be obtained at increased yields.

## Conclusion

It was found that dual emission of GSH/FA-based products does not originate from a single CD emitter but rather from a mixture of physically separate blue-emissive CDs and red-emissive porphyrin compounds. We demonstrate an easy way to separate the two synthesis compounds without the need for time-consuming dialysis. Systematic variations in synthesis parameters resulted in dramatically different product distributions, which can be explained via a simple model in which a porphyrin-forming reaction pathway is in direct competition with a carbonization pathway forming CDs. By understanding the nature of the system, we are now able to steer the synthesis process toward the desired product, which opens up a cheaper and more environmentally friendly synthesis route to CDs, water-soluble porphyrin, and mixed systems. With such control over the GSH/FA-based synthesis, the obtained CD system is another step closer to its application. The large shift between the Soret and Q absorption band(s) of the water-soluble porphyrin makes it, for example, interesting for applications in dye-sensitized solar cells.^54^ Finally, our findings motivate the detailed characterization and purification of other dual-fluorescence CD systems both from synthetic and natural carbon sources.^40^

## Supporting Information Available

Table with details of the synthesis reagents and the solvothermal treatment temperature, time, and additives; photoluminescence emission spectrum of dialysate passed through a 3.5 kDa and 12.4 kDa membrane and products inside a 12.4 kDa membrane of GSH-FA-3h dialyzed for a week and photoluminescence emission spectrum of a glutathione and formamide solution heated at *T* = 160 °C for 1 min; XRD pattern of samples GSH-FA-3h and GSH-FA-18h after dialysis with 3.5 kDa membrane and deposition on the glass coverslip; tentative assignment of observed IR bands; in situ photoluminescence emission and offset UV–vis absorbance spectra of 100 mg glutathione in 5 mL formamide solution during the heating up to 180 °C phase and held at this temperature sampled at various times; photoluminescence emission spectrum of reaction products of the solvothermal treatment of glutathione in different solvents; photoluminescence emission spectrum of the reaction products of the solvothermal treatment in formamide of single and pairs/triplets of amino acids in glutathione; and transmission electron microscopy images of GSH-FA-3h with self-assembled porphyrin and carbon dot clusters dried on a TEM grid, and optical microscopy images of dendritic self-assembly of porphyrin observed in dried GSH-FA-3h sample (PDF)
