# Supplementary material for: Dual Fluorescence in Glutathione-Derived Carbon Dots Revisited
Source: J Phys Chem C Nanomater Interfaces. 2022 Jan 26;126(5):2720–7. doi: 10.1021/acs.jpcc.1c10478 (PMC8842246; doi:10.1021/acs.jpcc.1c10478)
Supplement: Supplementary file 1 — jp1c10478_si_001.pdf [file jp1c10478_si_001.pdf]

Dual Fluorescence in Glutathione-Derived Carbon Dots Revisited

*Yadolah Ganjkhanlou,‡ J.J. Erik Maris,‡ Joris Koek, Romy Riemersma, Bert M. Weckhuysen, and Florian Meirer**

Inorganic Chemistry and Catalysis, Debye Institute for Nanomaterials Science, Utrecht University, 3584 CG Utrecht, The Netherlands

* Corresponding author e-mail address: f.meirer@uu.nl

Table S1. Details of the synthesis reagents and the solvothermal treatment temperature, time, and additives.

Sample	GSH (g)	Solvent	Solvent (mL)	T (˚C)	Time	Additives
GSH-FA-3h	0.3	FA	10	180	3h	-------------
GSH-FA-18h	0.3	FA	10	180	18h	-------------
GSH-l-FA-3h	0.15	FA	10	180	3h	-------------
GSH-h-FA-3h	1.0	FA	10	180	3h	-------------
GSH-DMF-3h	0.3	DMF	10	180	3h	-------------
GSH-H2O-3h	0.3	H_2O	10	180	3h	-------------
GSH-FA-5min-Acidic	0.1	FA	5	180	5 min	1 mL HCl(37%)
GSH-FA-5min	0.1	FA	5	180	5 min	-------------
GSH-FA-5min-Basic	0.1	FA	5	180	5 min	0.2 g NaOH

Table S2. Tentative assignment of observed IR bands.

Band position cm^{-1}	Possible assignment	Description and Ref.
>3300	O-H stretching	Broad band due to H bonding, [1-3]
3320	N-H stretching	[1-3]
3185	C-H stretching	[1-3]
2900	C-H stretching	[1-3]
2885	C-H stretching	[1-3]
1673	C=O or C=N stretching	[1-3]
1591	C=C stretching	[1-3]
1387	C-H bending	[1-3]
1309	C-N stretching	In aromatic amines, [1-3]
1098	C-O stretching	1-3
1044	S=O	1
950	C=C bending	1-3
795	C-H bending	1-3

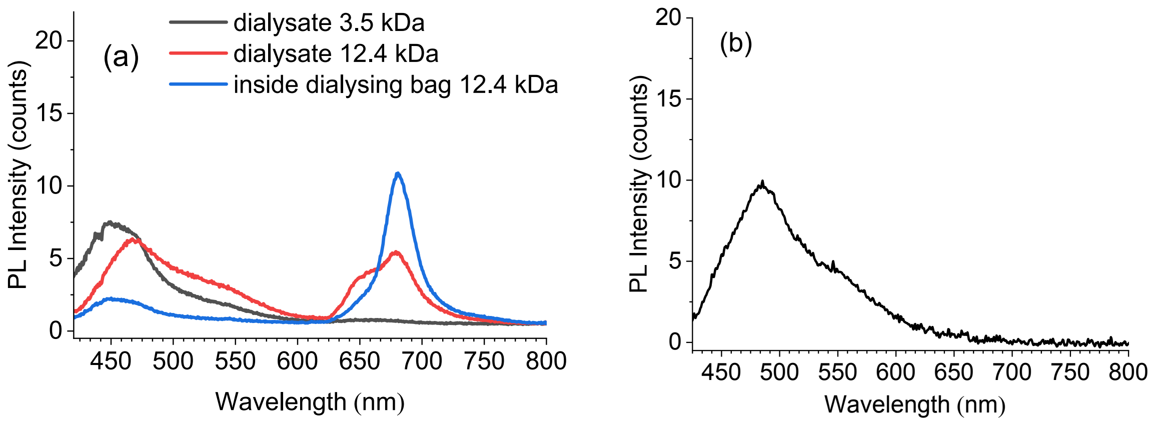

Figure S1. a) Photoluminescence emission spectrum of dialysate passed through a 3.5 kDa and 12.4 kDa membrane and products inside a 12.4 kDa membrane of GSH-FA-3h dialysed for a week. b) Photoluminescence emission spectrum of a glutathione and formamide solution heated at T = 160˚C for 1 min.

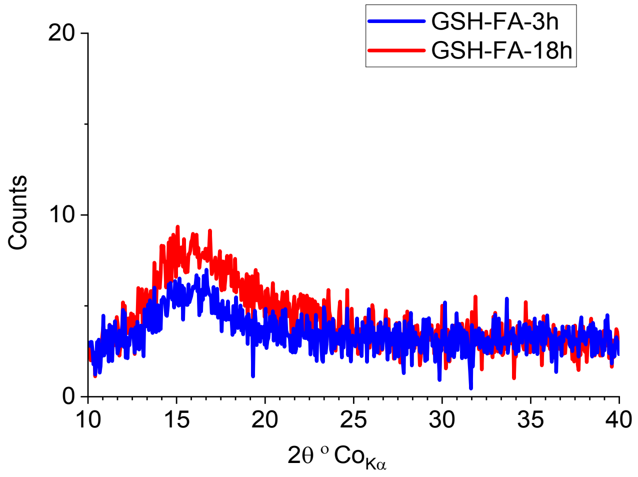

Figure S2. The X-ray diffraction pattern of sample GSH-FA-3h and GSH-FA-18h after dialysis with 3.5 kDa membrane and deposition on glass cover slip.

The X-ray diffraction patterns of the prepared carbon dots have a broad diffraction line at $2\theta CoK\alpha$ of 16° (d = 5.72 Å). Carbon dots synthesized with bottom-up approaches (especially those synthesized below 200°C) usually are near-amorphous in nature [4], which results in broadening of the diffraction line. The observed d spacing of 5.72 Å is larger than the typical 002-diffraction line of graphene (observed at $d \approx$ 3.38 Å). Enlarged interlayer spacing of CDs is reported to be due to the presence of different functional groups in CDs (especially oxygen-containing ones).[5]

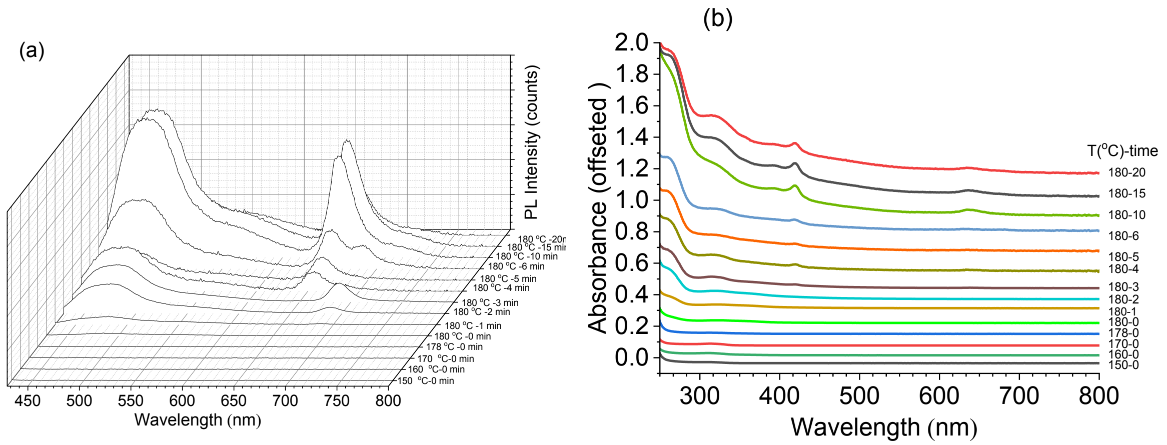

Figure S3. In-situ a) photoluminescence (PL) emission and b) offset UV-Vis absorbance spectra of 100 mg glutathione in 5 ml formamide solution during the heating up to 180˚C and hold at this temperature sampled at various times. The formation of porphyrin occurs mostly in the first 10 minutes of the reaction, after the onset of the formation of blue-emitting fluorophores.

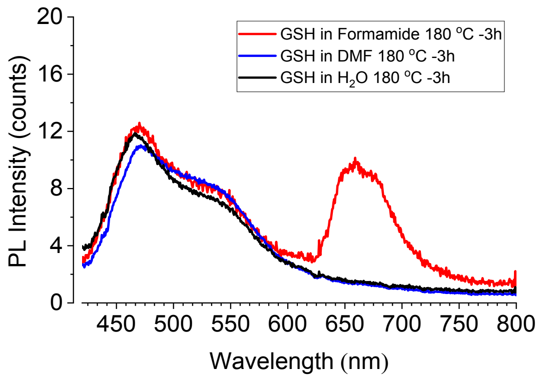

Figure S4. Photoluminescence emission spectrum of reaction products of the solvothermal treatment of glutathione in different solvents. Only glutathione in formamide yields photoluminescence emission in the 600–750 nm regime.

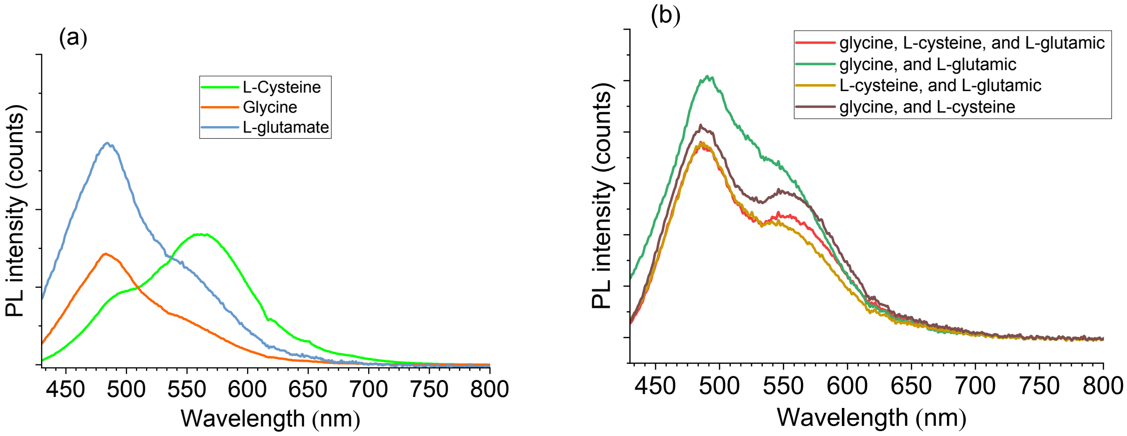

Figure S5. a–b) Photoluminescence emission spectrum of the reaction products of the solvothermal treatment in formamide of single (a) and pairs/triplets of (b) amino acids in glutathione. Even though these amino acids make up the glutathione peptide, they do not yield emission in the 600–750 nm regime, indicating that no porphyrin is formed. The concentration of all reagents in formamide was 150 mM in this experiment.

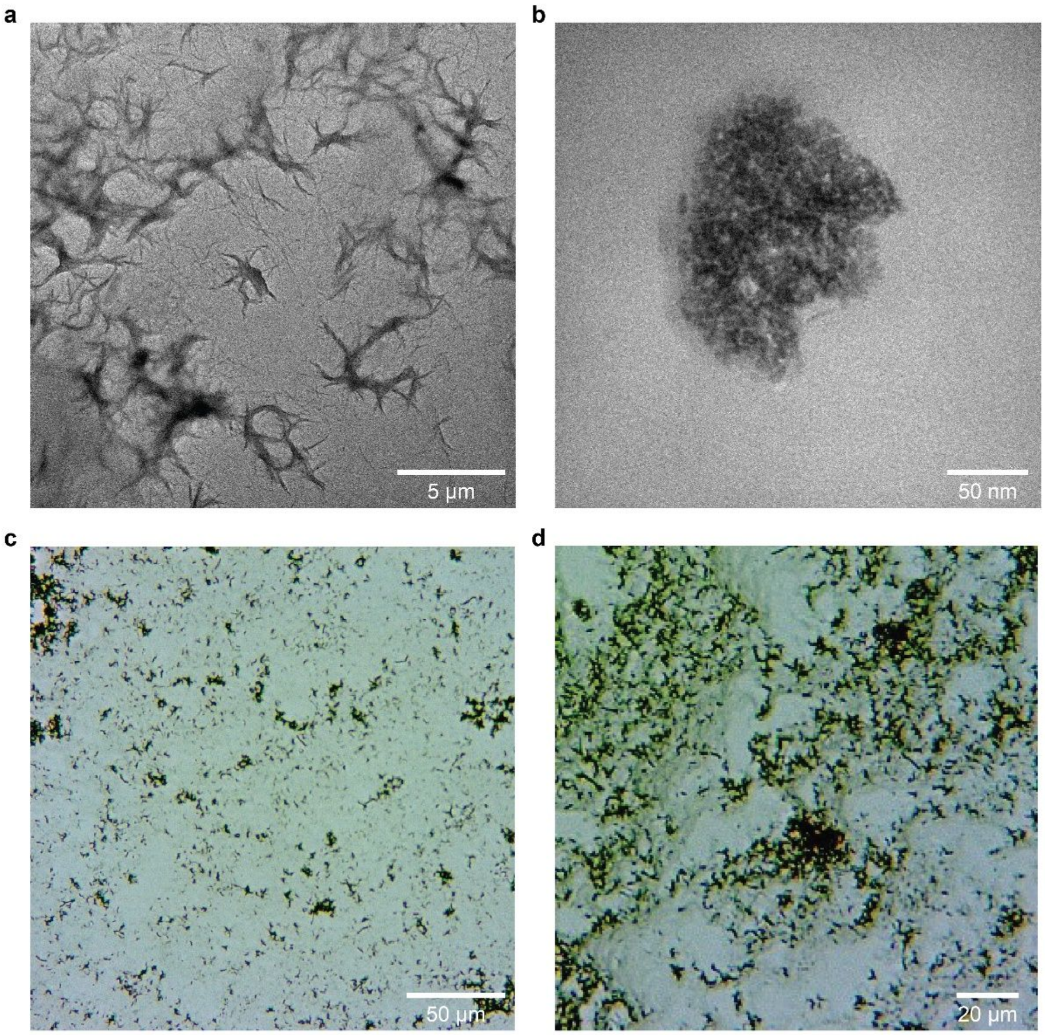

Figure S6. a–b) Transmission electron microscopy (TEM) images of GSH-FA-3h with self-assembled porphyrin (a) and carbon dot clusters (b) dried on a TEM grid. The carbon dots are visible at high magnification. The size of the carbon dots is around 3–5 nm, which is in agreement with AFM topography measurements (Fig. 3d). c–d) Optical microscopy images of dendritic self-assembly of porphyrin observed in a dried GSH-FA-3h sample. The green color of dendritic structures is in line with the measured UV-Vis absorption spectrum of the porphyrin derivatives,

which verifies that these self-assemblies are related to porphyrin. Indeed, porphyrin is a two-dimensional macromolecule, and a majority of its derivatives are hard to crystallize and tend to form amorphous assemblies.[6-7]

REFERENCES

1. Lin-Vien, D.; Colthup, N. B.; Fateley, W. G.; Grasselli, J. G., *The Handbook of Infrared and Raman Characteristic Frequencies of Organic Molecules*; Elsevier, 1991.

2. Mitchell, R. T., Synthesis of Water Soluble Porphyrins and Their Applications. **2016**.

3. Namitha, P. P.; Saji, A.; Francis, S.; Rajith, L., Water Soluble Porphyrin for the Fluorescent Determination of Cadmium Ions. *Journal of fluorescence* **2020**, *30*, 527-535.

4. Xiong, Y.; Schneider, J.; Ushakova, E. V.; Rogach, A. L., Influence of Molecular Fluorophores on the Research Field of Chemically Synthesized Carbon Dots. *Nano Today* **2018**, *23*, 124-139.

5. De, B.; Karak, N., A Green and Facile Approach for the Synthesis of Water Soluble Fluorescent Carbon Dots from Banana Juice. *Rsc Advances* **2013**, *3*, 8286-8290.

6. Battersby, A. R., Tetrapyrroles: The Pigments of Life. *Natural product reports* **2000**, *17*, 507-526.

7. Goldberg, I., Crystal Engineering of Nanoporous Architectures and Chiral Porphyrin Assemblies. *CrystEngComm* **2008**, *10*, 637-645.
